# Causes, prevention, and interventions regarding classroom disruptions in digital teaching: A systematic review

**DOI:** 10.1007/s10639-021-10795-7

**Published:** 2021-11-12

**Authors:** Pierre Meinokat, Ingo Wagner

**Affiliations:** Centre of Teacher Training, Karlsruhe Institute of Technology, Kaiserstraße 12, Geb. 20.52, 76131 Karlsruhe, Germany

**Keywords:** Classroom Disruptions, Digital Teaching, Systematic Review, Classroom Disturbances, Prevention, Interventions

## Abstract

Digitization and the Sars-CoV-2 pandemic are accelerating the use of digital tools in teaching. Therefore, this systematic literature review offers an overview of international studies with a particular focus on classroom disruptions and their causes, as well as on prevention and intervention strategies in digital settings. Selecting out of over 700 published articles from the last 20 years, the results show that, although the research on classroom management in general is numerous, the connection between digitization and classroom disruptions has received little attention so far. Studies of different methodological orientations have been conducted, but strongly teacher-focused. Also, there are conceptual inaccuracies leading to a variety of different findings and interpretations. Thus, this article provides a definition of the term digital teaching and critically discusses the classification of new findings, their emplacement in existing research, as well as their potential to expand existing models. Furthermore, the results summarize causes of disruptions in digital teaching, their possible prevention and intervention strategies.

## Introduction

Classrooms without disruptions are desirable, yet utopic. Thanks to Kounin (1970), the field of classroom management has been researched greatly in the last 50 years, and findings show that handling disruptive classroom situations is part of classroom management (Durak & Saritepeci, 2017; Egeberg et al., 2016; Kubat & Dedebali, 2018; Moltudal et al., 2019). Moreover, “effective classroom management has been identified as a key predictor of student success” (Marquez et al., 2016, p. 88). However, the emerging field of digital teaching has seldom been studied regarding disruptions.

Yet, information and communications technology (ICT) can be found in various parts of everyone’s daily life nowadays. For example, in the early 2000s, the first widely used mobile phones were produced, personal computers entered private households, the internet became accessible, and social media platforms like Facebook and YouTube were made available. Nowadays data is processed digitally at work, smart phones and tablets are more and more used on every educational level and modern science is based on international communication via technology. Undoubtedly, ICT has grown in importance and is receiving more and more attention. The penetration of educational areas with ICT and digitization in general seems inevitable, especially since the outbreak of the Sars-CoV-2 pandemic in 2020. Global education is being forced toward digitization faster than ever before and the use of ICT plays a crucial role in this. It is worth mentioning here that the use of ICT in education is important on different levels. Information systems like (e.g.) internet forums and social networks happen to get more and more integrated in daily educational practice as well as communication systems (like smartphones) and computer systems including their hardware (PCs, Laptops, etc.) and software (apps, learning platforms, etc.). While subjects like computer science are inevitably connected to ICT, other subjects are increasingly using the advantages of digital tools. With a first brief view on the field of classroom disruptions, the increasing usage of ICT evolves potential problems in a variety of areas. For example: problems with the school network, data protection for students’ and teachers’ data, etc. All these topics are located in one or more areas of information and communication technologies and therefore, this review will refer to all these different forms as ICT. All forms of education from primary education to university level are affected. International research focuses on education in digital settings in order to improve knowledge of teachers and students who are forced away from traditional face to face education. As part of this research, this review will contribute to attempts to improve the education of future teachers and will summarize information needed to train teachers on their classroom management skills.

The importance of a well-developed digitization (culture) and the proper use of ICT in teaching and learning had been highlighted around the world by governments and related institutes (e.g., Kultusministerkonferenz [KMK], 2017; U.S. Department of Education, 2017) even before this global incident. Although its importance has been recognized, current research “does illuminate the slow pace at which classroom management research has entered the digital age” (Cho et al., 2020, pp. 8–9). Scientific research is responsible for accompanying this “digital revolution” (Collins & Halverson, 2018, p. 1), and there is a need to enhance already existing research on classroom management and digitization in education while creating a new foundation for future research in digital teaching. Special attention should be paid to disruptions in digital teaching and how to prevent or deal with them in order to make such teaching as effective as possible. As a part of this task, this review aims to reveal the current state of international research in the field of classroom disruptions in digital settings and to discover possible research gaps to enhance the future quality of digital teaching.

## Framework

Two main concepts appear when focusing on the objective of this review. First, there is the concept of *classroom disruptions*. This is defined as “behavior a reasonable person would view as being likely to substantially or repeatedly interfere with conduct of a class” (Stockton University, 2001, p. 1). The mentioned behavior can further be described as “any behavior that interferes with teaching and learning” (Franken, 2020, p. 445). However, current research is missing sorts, types, or patterns of classroom disruptions besides problematic student behavior. A deeper look into existing research reveals that classroom disruptions are seldom investigated on their own and are mostly found in a contextual setting. This setting is widely known as *classroom management* and is much more researched than classroom disruptions alone. It was Kounin (1970) who conveyed the term classroom management to a wider audience. It was defined as “the actions teachers take to create an environment that supports and facilitates both academic and social-emotional learning” (Evertson & Weinstein, 2006). During the last 50 years of research into classroom management, scientists have come up with numerous models regarding what it is and what it includes, in contrast to research on the specific sub-area of classroom disruptions. As Balli (2011) mentioned, three models have appeared most often in teacher education research. First, the assertive discipline model (Canter, 2009) was criticized for its more traditional (student and teacher) behavior approach and was updated with more emphasis on establishing a positive classroom climate through rules and procedures (Balli, 2011, p. 246). Second, Kounin’s (1970) withitness and group management model demanded teachers to remain aware and revealed the need to multitask and the risk of focusing on one student for too long (Balli, 2011, p. 246). Third, the choice theory model by Glasser (1986) advised teachers to be lead managers in a democratic classroom rather than boss managers controlling students (Balli, 2011, p. 246). While these models focused on a more constructional view of teachers’ behavior, recent research has shifted its focus to categories of classroom management in daily practice (Greenberg et al., 2014).

Three different reviews, Simonsen et al. (2008), Oliver et al. (2011), and Epstein et al. (2008), together included over 150 different studies of classroom management from the last 60 years. “Despite the wide variation in research citations in these sources, there is congruence in their findings on essentially five strategies for classroom management” (Greenberg et al., 2014, p. 3). These big five are as follows: rules, routines, praise, misbehavior, and engagement (Greenberg et al., 2014, pp. 3–4). Misbehavior, as an essential part of classroom management, leads to disruptions in classrooms and therefore “harm[s …] the teaching/learning process” (Maddeh et al., 2015, p. 144). This review will search for disruptive situations within a learning process to have a wide overview of possible actions that can be understand as classroom disruptions. To do this in a systematic way, mentioned models and definitions will be used. Yet, what all models and definitions have in common is that they lack a connection to a digital setting.

The second main concept of this review is *digital teaching*. While digitization seems to be a widespread social topic, a definition of digital teaching is still lacking. First, as a temporary conceptual definition, this review concentrates on the two individual terms *digital* and *teaching*. “Nowadays the term digital is used instead of such previously used terms as information and communication technology (ICT) or information technology when talking about technology-related skills” (Ilomäki et al., 2016, p. 656). Based on this definition this review will understand the term *digital* related to any kind of technology supported settings. The second term *teaching* on the other side has a wide field of definitions that can be found in numerous publications. For the purpose of this review the authors decided to use the definition of the Stellenbosch University (2021): “Teaching can be defined as engagement with learners to enable their understanding and application of knowledge, concepts and processes.” Classes are mentioned as a common place for this engagement. In combination with and without the term *digital*, various forms of settings can be found: face-to-face teaching in a non-digital setting, face-to-face teaching supported by digital tools or “virtual classroom[s]” (Kennedy-Clark, 2011; Park et al., 2019). These different kinds of classes were often considered from a learner’s perspective where it is not about the teaching and more about the learning aspect. This is supported by the fact that, in contrast to digital teaching, digital learning is described by several terms, some of which are used synonymously: “digital learning” (Harju et al., 2019), “e-learning” (Basak et al., 2018; Kriesen, 2011), “blended learning” (Akhtar et al., 2017; Mason, 2005), and “mobile learning” (Burden et al., 2019; Charles, 2012). This shows the need for a clearer definition of digital teaching, which will be addressed through this review.

When faced with disruptive situations in a digital setting, two possible, timewise-different points of view appeared: the time before, called prevention, and the time during/after a disruption, when an intervention may be applied. To create a greater understanding of each complete situation, research has often looked at these two periods simultaneously (Cerezo et al., 2017; Walonoski & Heffernan, 2006). Intervention strategies were often related to teachers’ behavior (Gebbie et al., 2012), while prevention strategies often searched for causes (Handley et al., 2016). To generate a complete overview of the current literature on classroom disruptions in digital teaching, this review covers articles about causes, prevention, and interventions. To date, despite the high relevance resulting from the increase in digital teaching, no such overview exists.

## Research questions

To create an overview, discover problems, and find research gaps, this systematic literature review elaborated multiple research questions (RQs):

- RQ 1: Which terminologies can be found in research literature to describe digital teaching and the disruptions within it?
- RQ 2: What are the methodological approaches used in previous research on the subject of disruptions in digital teaching?
- RQ 3: How does research systematize disruptions in digital teaching?
- RQ 4: What are the causes of disruptions in digital teaching and what prevention and intervention strategies exist to deal with them? To answer this question properly, three sub-questions were generated:
- RQ 4.1: What are the causes of disruptions in digital teaching?
- RQ 4.2: How can disruptions in digital teaching be prevented?
- RQ 4.3: What intervention strategies regarding disruptions in digital teaching are addressed in the research literature?

## Methods

This review used the following databases: *Education Resources Information Center (ERIC)*, *Academic Search Ultima (*via* EBSCOhost),* and *Web of Science.* All the databases included pedagogical literature as well as literature related to the computer scientifical/technological field. This led to a higher output of potential studies to review and decreased the chance of missing publications in this field of research. Since the number of investigations on digitization has grown over the last decades, only studies published in the last 20 years were included. Also, only English-language, peer-reviewed, study-based articles were integrated to maintain a high and international scientific standard. The general review procedure was based on the PRISMA statement of Moher et al. (2009).

For the literature research, a search string was developed based on the current scientific point of view, as explained in the framework earlier. Based on the terminologies found in the classroom management models and the big five, word clusters based around (mis-)behavior, discipline, classroom, and disruption were created for the concept of classroom disruptions. Due to the missing definition of digital teaching, closely related terms, like learning and education, were connected to analogies for the term digital, like electronic and virtual. In combination, this led to this search string (which was modified in terms of syntax to fit in the different databases):

("behavior management" OR "behaviour management" OR "behavior problems" OR "behaviour problems" OR "behavior referrals" OR "behaviour referrals" OR "discipline policy" OR "discipline referrals" OR "classroom dilemmas" OR "classroom management" OR "disruptive behavior" OR "disruptive behaviour" OR "school discipline" OR "student behavior" OR "student behaviour" OR "misbehavior" OR "misbehaviour") AND ("e-learning" OR "digital learning" OR "digital education" OR "virtual classroom" OR "electronic learning" OR "blended learning").

Because of problems in converting the search string for different databases, truncation was avoided. This search string created a total of 724 hits. Duplicates were eliminated using the reference management software Citavi, leaving 705 articles divided between the databases as follows:

- ERIC: 453 articles
- Academic Search Ultimate (via EBSCOhost): 228 articles
- Web of Science: 24 articles

These 705 articles were screened based on their titles and abstracts. During the title screening, another 12 articles were found to be duplicates, so 693 articles passed to the title and abstract screening. Exclusion criteria were set to eliminate articles that did not match the scope. Due to lack of a definition of digital teaching, the abstracts were searched for possible digital or digital supported executions of teaching and a consideration of disruptions inside or related to this setting. Therefore, we focused on the main aspects that can be found in the title of this review as well: Are there causes of classroom disruptions mentioned? Can prevention or intervention strategies be found/expected to be part of the article? Does the title/abstract at least mention classroom disruptions or synonymously understood terms? Is there some sort of digital setting acknowledgeable in the title or the abstract? Articles not fitting this purpose were excluded. Studies located in a non-school setting, like advanced technical training for jobs or medical education, were excluded as well. Often connected to a medical point of view were behavioral issues based on emotional and behavioral disorders, attention-deficit/hyperactivity disorder (ADHD), and likewise. Since this review focuses on the educational context, medical contexts were also eliminated. Also, the term *cheating* was found several times during the screening but was not included in this review because of its different intention of use. These criteria, in combination with the organizational framework (a study, published in English, etc.), were applied by the authors and a co-worker to create a reliable outcome of 50 articles left for a selective review. The final coincidence rate of 98% was reached through 3 screening steps. The researchers and co-worker screened the articles independently from each other, and their results were shared after screening the first 50, then half of, and finally all the abstracts. General problems in understanding different designations were discussed, and a uniform understanding, which will be part of the discussion section of this review, was created during these iterations. This procedure left 50 articles for the next step.

During the selective review, the articles were reviewed based on their findings. In general, the same inclusion criteria were used on the selective review as for the title and the abstract earlier in the process. The authors focused on the described methods, the results and conclusion out of the results found in the article. This more selective procedure allowed to scan all 50 articles in more detail and to decide if they fit in the purpose of this systematic review. Studies that did not meet the given criteria, as well as those that were not studies were excluded. This reduced the results down to a final total of 16 articles, that deal at least partially with causes, prevention and intervention strategies on disruptions in the context of digital or digital supported teaching.

**Fig. 1:**
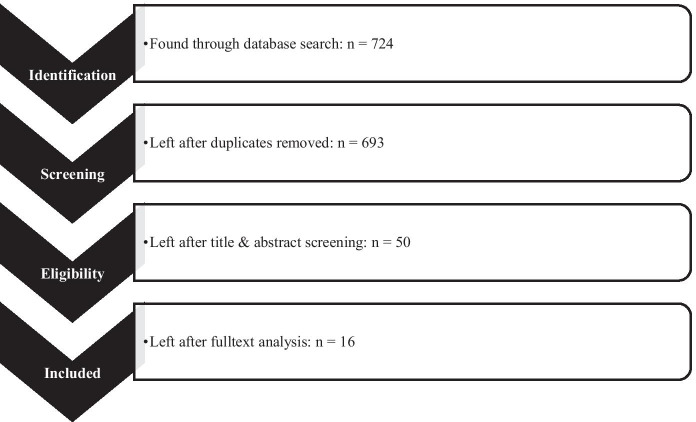
Procedure according to the PRISMA statement (Moher et al., 2009)

These 16 articles have been fully analyzed. Therefore, we have read the full texts several times with a strict focus on a specific research question. With the help of the knowledge organization functions of the reference management software Citavi, we were able to generate and collect answers to the research questions depending on the orientation and detail of the respective article. Table 4 (Appendix 1) shows the entire collection of results. Figure 1 illustrates the reviewing process.

## Results

All 16 reviewed articles are listed in Table 4 (Appendix 1). The results of this review will be presented according to the research questions.

### RQ 1: Which terminologies can be found in research literature to describe digital teaching and the disruptions within it?

The results with regard to this research question will be shown separated in terms of the general term *digital teaching* and the *disruptions* therewithin.

#### Digital teaching

With respect to the terms applied to *digital teaching*, the authors used different word cluster concepts, which varied in terms of their precision. While some of them mentioned very general explanations, like “internet” (Boyaci, 2010, p. 208), “online” (Hummel et al., 2015, p. 670; Li & Titsworth, 2015, p. 41), “software” (Boyaci, 2010, p. 213), or “tools” (Charles, 2012, p. 10), others decided to name the exact tools, like “cell phones” (Charles, 2012, p. 4; Storch & Juarez-Paz, 2019, p. 12), “laptop” (Rosen & Beck-Hill, 2012, p. 228), and “video” (Kurz & Batarelo, 2010, p. 46). The majority of the researchers opted for grouping the terminology.

Some research held on to the term *digital* and combined it with other terms to create word clusters for digital teaching. This let to designations like “digital learning” (Blundell et al., 2019, p. 1), “digital […] system” (Homer et al., 2018, p. 137), “digital technologies” (Blundell et al., 2019, p. 1), or “social digital networks” (Charles, 2012, p. 4).

Another group of terms, like “computer based” (Muir et al., 2013, p. 1), “data-based” (Bruhn et al., 2020, p. 1), and “web based” (Boyaci, 2010, p. 208), was more related to computers and electronic data processing.

The field of virtual reality contained terms such as “mixed reality” (Judge et al., 2013, p. 88), “virtual classroom” (Muir et al., 2013, p. 1), “virtual environment” (Judge et al., 2013, p. 92; Muir et al., 2013, p. 4), and “virtual technology” (Judge et al., 2013, p. 88).

The approach of not mentioning the term *digital* directly but of instead applying connections to the word *technology* was used, for example, “educational technology” (Rosen & Beck-Hill, 2012, p. 226), “Information and Communication Technology (ICT)” (Heitink et al., 2017, p. 96; Moltudal et al., 2019, p. 80), “mobile technologies” (Charles, 2012, p. 14), and “technology based” (Bruhn et al., 2020, p. 1).

Table 1 shows the division of the conceptual groups. Detailed information on which terminology can be found in which article can be seen from Table 4 in the Appendix 1. Observing the amount of different designations used, it became clear that there is still no standardized terminology when it comes to digital teaching.

**Table 1:** Taxonomy—groups of terms for digital teaching

digital teaching (n = 33)	technology related (n = 12)	- “technology” (n = 3) - “ICT” (n = 2) - “digital technologies” (n = 1) - “educational technology” (n = 1) - “mobile technologies” (n = 1) - “technical” (n = 1) - “technology-based” (n = 1) - “technology integration” (n = 1) - “virtual technology” (n = 1)
	general explanations (n = 5)	- “online” (n = 2) - “internet” (n = 1) - “software” (n = 1) - “tools” (n = 1)
	virtual related (n = 5)	- “mixed reality” (n = 1) - “virtual classroom” (n = 1) - “virtual environment” (n = 1) - “virtual technology” (n = 1) - “virtual worlds” (n = 1)
	digital related (n = 4)	- “digital badges-and-points” (n = 1) - “digital learning” (n = 1) - “digital networks” (n = 1) - “digital token point system” (n = 1)
	exact tools (n = 4)	- “cell phones” (n = 2) - “laptop” (n = 1) - “video” (n = 1)
	computer related (n = 3)	- “computer based” (n = 1) - “data based” (n = 1) - “web-based” (n = 1)

#### Disruptions

In accordance with the digital teaching terms, those describing the types of disruptions could also be systematized into subgroups.

By far the biggest group of terminologies found was connected to the term *behavior*. Combinations like “behavior disruptions” (Judge et al., 2013, p. 95), “behavior management” (Judge et al., 2013, p. 88), “behavioral expectations” (Storch & Juarez-Paz, 2019, p. 12), “challenging behavior” (Bruhn et al., 2020, p. 1), “classroom behavior management” (Muir et al., 2013, p. 1), “disruptive or unengaged behavior” (Judge et al., 2013, p. 90), “inappropriate behaviors” (Ho, 2004, p. 386), “incompatible behavior” (Judge et al., 2013, p. 88), “misbehaviors” (Li & Titsworth, 2015, p. 41), “negative behavior” (Homer et al., 2018, p. 140), and “problem behaviors” (Bruhn et al., 2020, p. 4) were mentioned.

Some authors used wider terms, such as “being off-task” (Homer et al., 2018, p. 141) or “non-academic activities” (Moltudal et al., 2019, p. 81), or declared them to be a “crime” (Moltudal et al., 2019, p. 84), when talking about disruptive issues.

With the terms “rule-breaking” (Charles, 2012, p. 8) and “rule violations” (Baker et al., 2016, p. 22), researchers focused more on the advantages and problems of rules. This can also be connected to “disciplinary issues” (Rosen & Beck-Hill, 2012, p. 234).

The complexity of classroom management was found in terminological combinations as well: “classroom behavior management” (Muir et al., 2013, p. 1), “classroom management dilemmas” (Hummel et al., 2015, p. 670), and “typical classroom management situations” (Kurz & Batarelo, 2010, p. 49). As Muir et al. (2013) showed, the boundaries were sometimes blurry.

Table 2 shows the division of the conceptual groups. Detailed information on which terminology can be found in which article can be seen from Table 4 in the Appendix 1.

**Table 2:** Taxonomy—groups of terms for classroom disruptions

classroom disruption (n = 29)	behavior related (n = 19)	- “inappropriate behaviors” (n = 3) - “problem behavior” (n = 3) - “behavior management” (n = 2) - “behavioral expectations” (n = 1) - “behavioral interventions” (n = 1) - “behavior supportive adaptive teaching” (n = 1) - “challenging behavior” (n = 1) - “classroom behaviours” (n = 1) - “disruptive behavior” (n = 1) - “incompatible behavior” (n = 1) - “misbehavior” (n = 1) - “negative behavior” (n = 1) - “off-task behavior” (n = 1) - “student behaviours” (n = 1)
	rules and disciplinary (n = 4)	- “breaking rules” (n = 1) - “disciplinary issues” (n = 1) - “rules violations” (n = 1) - “students without discipline” (n = 1)
	classroom management related (n = 3)	- “classroom behavior management” (n = 1) - “classroom management dilemmas” (n = 1) - “typical classroom management situations” (n = 1)
	wider tasks (n = 3)	- “crime-control” (n = 1) - “disruptions” (n = 1) - “non-academic use” (n = 1)

### RQ 2: What are the methodological approaches used in previous research on the subject of disruptions in digital teaching?

Since the research field has still not been explored much, the methods used in the research studies vary. Table 4 (Appendix 1) shows the kind of research design, the number of participants, and the digital tools used in each article.

Of the 16 articles considered for this review, 7 articles (Baker et al., 2016; Blundell et al., 2019; Boyaci, 2010; Charles, 2012; Kurz & Batarelo, 2010; Muir et al., 2013; Storch & Juarez-Paz, 2019) made use of a purely qualitative approach, and 3 (Heitink et al., 2017; Ho, 2004; Judge et al., 2013) chose a quantitative one, leaving 6 studies that utilized a mixed-methods approach (Bruhn et al., 2020; Homer et al., 2018; Hummel et al., 2015; Li & Titsworth, 2015; Moltudal et al., 2019; Rosen & Beck-Hill, 2012). In terms of the qualitative articles, content analyses based on written texts and semi-structured interviews were applied the most. Questionnaires appeared in most of the quantitative articles. As the mixed-methods approach implies, the use of research methods from both areas had multiple goals, for example, to “explore and discover (qualitatively), and then test and confirm (quantitatively)” (Moltudal et al., 2019, p. 84).

The numbers of participants in each study differed as well. The smallest groups were n = 6 by Blundell et al. (2019) and Judge et al. (2013). By far the biggest number of participants was found in the quantitative (second) part of Moltudal et al.’s research (2019) (n = 2579). It is worth mentioning that every study included teachers somehow. Practicing teachers were part of 10 studies (Blundell et al., 2019; Bruhn et al., 2020; Charles, 2012; Heitink et al., 2017; Ho, 2004; Homer et al., 2018; Li & Titsworth, 2015; Moltudal et al., 2019; Rosen & Beck-Hill, 2012; Storch & Juarez-Paz, 2019), and 6 studies mentioned beginning/pre-service teachers (Baker et al., 2016; Boyaci, 2010; Hummel et al., 2015; Judge et al., 2013; Kurz & Batarelo, 2010; Muir et al., 2013). Combined with this, in 5 studies, students were also a part of the research design (Bruhn et al., 2020; Homer et al., 2018; Li & Titsworth, 2015; Moltudal et al., 2019; Rosen & Beck-Hill, 2012).

The digital tools used differed just as much as the school levels at which they were used. Table 3 gives an overview what form of tool was used for which level of education. To make it easier to compare, the levels of education were divided into primary education (like elementary school), secondary education (middle and high school) and university level. If mentioned in the article, the level of class (grade or year) is shown separately in an own column. As mentioned, the authors encountered the research field in a variety of ways, which is why there is no clear connection between digital tools used and the level of education. Furthermore, most studies do not specify their model of teaching. This is due to the fact that, on the one hand, the majority of the studies only partially deal with classroom disruptions and, on the other hand, a tendency towards recommendations for action can be recognized. This takes the focus away from the specific disruptive situation and is therefore no more analyzed in this review. In addition, an assignment to existing models of teaching, such as direct instructions, cooperative or project-based learning, in the articles can at best be speculative.

**Table 3:** Forms of digital tools used and their associated school level

Authors	Digital tool	Level of education	Level of class	Model of teaching
Baker et al. (2016)	Online forum used by pre-service teachers to discuss behavior	Primary and secondary education	Different, mostly unspecified level of class	Not given due to the fact that participants only describe their findings and strategies
Blundell et al. (2019)	Teachers’ opinion after transferring their own classes into digital classes	Primary and secondary education	Different, sometimes unspecified level of class	Varying teaching models, mostly not described, as the teachers changed their teaching style
Boyaci (2010)	Pre-service teachers’ opinions on web-based classroom management	Primary education	Unknown level of class	Not given
Bruhn et al. (2020)	Data-based, individualized interventions via app	Primary education	Unknown level of class	Face to face teaching, no more information
Charles (2012)	Cell-Phone usage in classes	Secondary education	10^th^ & 11^th^ grade	Face to face teaching, different teacher styles, no more information
Heitink et al. (2017)	Video-vignettes of teachers showing digital tools in classroom	Primary education	Different, unknown level of class	Face to face teaching, no more information given
Ho (2004)	Video-vignettes of teachers’ reactions to problematic behavior	Secondary education	Different, unknown level of class	Face to Face teaching, Various, not mentioned teaching models
Homer et al. (2018)	Comparing a digital badges and points system to a non-digital version used in classroom	Primary education	1^st^ to 4^th^ grade	Face to face teaching, double-period lessons for 1^st^ and 2^nd^ grade, single lessons for 3^rd^ and 4^th^ grade
Hummel et al. (2015)	Comparing an online game version to the non-digital version of a game about classroom disruptions	University level	3^rd^ year students	Half of the students worked in groups with the face to face version of the game, the other half worked in groups in the online version
Judge et al. (2013)	Virtual classroom-simulation	Secondary education	Unknown level of class	Online simulated face to face interaction with students
Kurz and Batarelo (2010)	Video-vignettes for training teachers	Primary and secondary education	Unknown level of class	Unknown teaching models in the video vignettes
Li and Titsworth (2015)	Online-classroom analysis and observation	University level	Unknown level of class	Online teaching, unknown further teaching model
Moltudal et al. (2019)	Study about teachers’ digital competences and their ICT usage	Secondary education	Unknown level of class	Not given
Muir et al. (2013)	Virtual classroom simulation	Primary education	Unknown level of class	Online simulated face to face frontal teaching
Rosen & Beck (2012)	Laptop environment for students	Primary education	4^th^ & 5^th^ grade	Face to face teaching, no more information
Storch & Juarez (2019)	Cell-Phone usage in classes	University level	various, unknown level of class	Various, no information given

Of the 16 studies, 2 approached the field of digitization on a more abstract level. Moltudal et al. (2019) took a deeper look into the digital competences of teachers in relation to the matter of classroom management and managing disruptive behavior. Li and Titsworth (2015), using an online classroom simulation as part of their study, developed the Student Online Misbehavior scale (SOMs).

### RQ 3: How does research systematize disruptions in digital teaching?

Only two research groups made an attempt to identify concrete disruptions within teaching and systemize them into categories. Ho (2004) delivered six types of problems, and for each of these, a video vignette was watched by participants. The types “were based on preliminary studies” (Ho, 2004, p. 378) in which teachers from Australia and Hong Kong responded to an open-ended question. This led to the identification of three “major types of problems[…:] learning motivation problems, disruptiveness in class, and inappropriate interpersonal behaviors” (Ho, 2004, p. 378). Teachers were asked about problem behaviors in general. Therefore, it can be assumed that these types of problems were not exclusively connected to digital teaching. This statement was supported by the fact that none of the examples given concerning the types of problems had a digital context or angle (Ho, 2004, p. 379).

This issue was taken up by Li and Titsworth (2015). They created the SOMs (Li & Titsworth, 2015, p. 41). In multiple studies, the researchers used misbehaviors reported by teachers who were teaching online classes to create four factors that influence misbehavior (including a total of 15 items – see Table 4 in the Appendix 1):

- “Seeking unallowed assistance”
- “Internet slacking”
- “Aggressiveness”
- “Lack of communication” (Li & Titsworth, 2015, p. 47)

The Researchers developed this scale through two separate studies. In the first study the purpose “was to inductively generate a typology of online student misbehaviors that could form an inventory for use in subsequent studies of online classes” (Li & Titsworth, 2015, p. 42). Results of the surveys generated twenty student misbehavior types which are summed up in the second study into the four factors of the SOMs by further exploration through additional surveys.

### RQ 4: What are the causes of disruptions in digital teaching and what prevention and intervention strategies exist to deal with them?

To look in more detail at the causes of disruptions, as well as at prevention and intervention strategies, three sub-questions were answered.

#### RQ 4.1: What are the causes of disruptions in digital teaching?

Of the 16 studies analyzed, 6 mentioned causes of disruptions within digital settings. As Blundell et al. (2019) and Storch and Juarez-Paz (2019) noted, digital tools themselves were sometimes a source of distraction. The possibilities offered by technological devices, like smartphones or tablets, seemed to also create opportunities to go off-task. As a major reason for this, teachers referred to a lack of self-regulation or self-discipline. “Teachers held students most responsible for displaying inappropriate behaviors (lack of […] self-discipline) and themselves as least responsible” (Ho, 2004, p. 386).

These issues in terms of self-regulation and self-discipline were observed in conjunction with another missing factor. In Boyaci’s (2010) study, multiple students “focused on limited communication” (Boyaci, 2010, p. 225), which did not appear to be an issue in non-digital, face-to-face education. The importance of good and detailed communication between teachers and students was demonstrated by the fact that multiple studies recommended a change in teaching style to more of a moderating and mentoring style (Boyaci, 2010; Moltudal et al., 2019).

However, it was not only the communication between students and their teachers that was observed to be important. As Charles (2012) showed, a possible cause of distraction was a lack of communication and coordination between teachers and officials. “Teachers enforce […] rules differentially” (Charles, 2012, p. 10). This made it difficult to predict what would be a distraction for each participant in a class.

#### RQ 4.2: How can disruptions in digital teaching be prevented?

Almost half of the reviewed articles (7 out of 16) gave an answer to RQ 4.2. Among these answers, “rule-setting” (Charles, 2012, p. 12) seemed to be the most advised prevention strategy. A clear understanding of what is appropriate and what is not was found to be essential for using digital tools in teaching.

Another strategy mentioned by Greenberg et al. (2014), not only useful in digital teaching, is creating routines: giving students and teachers the possibility of knowing what is coming next and how to act properly to avoid disruptions (Baker et al., 2016, pp. 26–28).

Charles (2012) suggested the development of meta-awareness. This includes teachers’ participation and underlines the aforementioned change of teaching style to a more moderating role.

Concrete strategies, like permanent and “individual communication” (Boyaci, 2010, p. 213), “active monitoring” (Storch & Juarez-Paz, 2019, p. 16), and “the use [of a] language of understanding” (Baker et al., 2016, p. 32), were also discussed.

However, for all the strategies it was important that teachers felt comfortable in terms of their teaching (Blundell et al., 2019, p. 9). It is important to mention that all the advice given was not exclusively limited to disruptions in digital teaching but could definitely be used within this setting.

Homer et al. (2018) showed that the use of a digital token system could lead to increased positive behavior and prevent problematic attitudes. This was not exclusively linked to problematic behavior within a digital setting, but it showed how parts of digitization themselves could be a prevention strategy. It also tried to use digitization as an opportunity to enhance existing strategies.

#### RQ 4.3: What intervention strategies regarding disruptions in digital teaching are addressed in the research literature?

Of the 16 articles reviewed, 3 provided an answer to this question. Storch and Juarez-Paz (2019) recognized the need to warn students to stay on-task or return to it when the usage of cell phones was observed to have led them off-task (Storch & Juarez-Paz, 2019, p. 14).

As part of their results, Bruhn et al. (2020) showed that an app could be used as an intervention strategy. Since students could monitor their rated behavior themselves, negative changes could be avoided by the students. Changes in student behavior were indirectly initiated by the teacher’s rating (Bruhn et al., 2020, p. 8). Like Homer et al.’s (2018) study, this showed a possible digital solution to a not exclusively digital problem.

Although Baker et al. (2016) gave a couple of possible intervention strategies for pre-service teachers, those strategies were not specifically designed to deal with disruptions in digital teaching.

## Discussion

As was already suspected, the pure quantity of terms found in answer to RQ 1 (Which terminologies can be found in research literature to describe digital teaching and the disruptions within it?) shows a significant need for further development. Since there is no standardized designation for teaching that uses digital tools or is based on them, it is inevitable to find multiple approaches to naming this construct.

The results of this review show that there are two general approaches: naming or grouping the tangible tools, for example, video, computer-based, digital system, or trying to combine these tools under the term *technology* and, optionally, combining it with an enhanced setting, like educational technology or information and communication technology. Technology is defined as “scientific knowledge used in practical ways” (Oxford University Press, 2020b). This does not explain what these practical ways are exactly and what knowledge is used for it. Assuming digital is defined as “using a system of receiving and sending information as a series of the numbers one and zero” (Oxford University Press, 2020a), it is conceivable that digital tools are required for the use of such technology.

On the other hand, along with the digital context, a clear understanding of the teaching setting varies from article to article. In general, three different types of teaching/learning capable of using digital tools were found among the studies: (1) purely online education in which teachers and students are only in touch online, (2) purely face-to-face education enhanced by digital tools and in which teachers and students are physically present at the same place at the same time, and (3) a mixed version called blended learning. This three-way division, in connection with the understanding of the term *digital* discussed above, allows an attempt at defining digital teaching:

Digital teaching is the generic term for online learning, digitally enhanced face-to-face learning, and blended learning, assuming that digital tools are used as technology to enable or support the respective form of teaching.

Another terminological problem in this review is associated with the term *disruption*. Results show that disruptions in teaching are often described as a sort of behavior, often associated with a related negative declaration, like inappropriate or problem behavior. However, the term *behavior* covers a wide area of understanding. Suler (2004), for example, sees behavior management in online learning as management to create a better learning output. Greenberg et al. (2014), on the other hand, show that the management of (disruptive mis-)behavior is indisputably connected to classroom management. Therefore, it is important to take a detailed look at the usage of the term behavior in order not to miss a possible connection to classroom disruptions, and then to separate those understandings that do not mention disruptions.

When mentioning classroom disruptions, it is noticeable that not all of the reviewed articles clarify what specific disturbing situations are declared as disruptions. Some authors give examples like “student told me he hated me” (Baker et al, 2016, p. 29), “students wanted to use digital technologies at times and in ways that were different to planned uses” (Blundell et al., 2019, p. 8), “daydreaming in class, not completing homework, talking in class, lesson disruption, bullying, and rudeness to the teacher” (Ho, 2004, p. 378), “talking out of turn” (Homer et al., 2018, p. 141), “pupil continues to disturb the lesson by insulting his peers” (Hummel et al., 2015, p. 672), “Seeking Unallowed Assistance, Internet Slacking, Aggressiveness, and Lack of Communication” (Li & Titsworth, 2015, p. 41) and “student’s discipline issues” (Rosen & Beck-Hill, 2012, p. 234). Other authors do not give explicit examples for disruptions but refer to (pre-service) teacher’s strategies (Baker et al., 2016; Judge et al., 2013; Muir et al., 2013) or perceptions and attitudes (Boyaci, 2010; Charles, 2012; Heitink et al., 2017; Kurz & Batarelo, 2010; Storch & Juarez-Paz, 2019). This illustrates understandings what classroom disruptions in detail contain, but the results are so divers that it is not possible to deduce which disturbances might be more relevant than others. Since this question is related to a subjective view it does not surprise that different teams of authors mention, that the severity of each disruption depends on the teacher’s perception (e.g. Charles, 2012; Moltudal et al., 2019; Storch & Juarez-Paz, 2019). This shows the teacher centered orientation of current research as well as a still missing analysis of classroom disruptions in detail.

While there are various definitions and models about classroom management, such things are rare for classroom disruptions alone. As mentioned, this topic can be found more embedded in classroom management models. This missing focus may often lead to a superficial perspective on disruptions as articles predominantly consider the misbehavior of students. If combined with the quite new field of digital teaching, this results in several approaches to discussion. On the one hand, there is need for a detailed model about classroom disruptions that includes more than just inappropriate student behavior. Teacher behavior as well as external factors, especially related to the newly used digital tools, can be causing disruptions as well. Bringing in the factor of digitization and the new use of ICT inside a classroom while disruptions occur, this creates multiple possible assumptions: Does ICT create disruptions that did not occur in former education? On the other hand, as Bruhn et al. (2020) show, the proper use of ICT can help teachers and students to handle disruptive situations better than without digital tools. This inevitably raises the question: Can digitization of teaching reduce disruptions? Scientific research on this field must face the fact that digital teaching has multiple dimensions and therefore, a complex answer can be assumed.

Since the use of ICT in daily life often creates new problems like missing connection, lack of electricity and so on, the growth of digital teaching may create a chance to shift the focus from classroom disruptions as mainly student-caused to a more open view of all possible triggers. In addition, a specific connection between the use of certain ICT and the occurrence of certain disruptions has been unexplored so far.

The models, causes, and prevention and intervention strategies found are rarely tied to a digital setting. This is not surprising keeping in mind that digital teaching is an enhanced form of teaching and is therefore likely to face problems and strategies that non-digital teaching has already considered. However, isolated results (for example, Homer et al. (2018)) show that there is a chance to use the strategies developed for non-digital teaching and enhance teaching in general with digital tools to take advantage of them and to deal with disruptions that occur in both digital and non-digital teaching. As disruptions are key in terms of classroom management, there is an urgent need to look deeper into opportunities like this.

In general, findings show that even after a structured selection process studies differ in their point of view on disruptions in digital teaching. Some studies focused on classes (and their disruptions) that are supported by digital tools, some studies investigated pure online (and therefore digital) classes and other studies used digital tools to evaluate disruptions in teaching. This realization raises the questions of an additional filtering of the selected studies. A more precise look would be able to offer more detailed results. Yet, it should be noted that further selection would also lead to an even smaller number of samples.

The results in relation to RQ 2, regarding the methodological approaches of previous research in terms of the subject of disruptions in digital teaching, show that the approaches in this field are methodologically broad. This may be explained by the fact that this field of research has still not been explored much. On the one hand, this has led to a pleasant variety of methodological approaches, but on the other hand, scientific comparison is therefore missing. In general, ongoing concepts shared between scientists and educators and developed by multiple researchers are missing as well. None of the reviewed studies was implemented as a longitudinal study, although approaches like that of Li and Titsworth (2015) used an iterative process to successfully develop a scale. Since this field of research is, compared to other parts of educational research, relatively young, future studies may fill this gap. This review provides a definition for digital teaching and clarifies the possibilities for enhancing existing models so that future research based on it seems promising.

Although the answers to RQ 3, how research systematizes disruptions in digital teaching, are the least elaborated on, they create a lot of potential for discussion. The SOMs derived by Li and Titsworth (2015) shows a first step to having a more detailed view of disruptions in digital teaching. Since the scale focuses on online teaching, the question arises of whether this scale might be transferable to other digital settings. During the development process, inputs given by teachers focused on online teaching, but transferring this to face-to-face or blended teaching seems practicable as well. It will be interesting to see if future scales in the area of digital teaching differ significantly from the SOMs.

Besides Li and Titsworth (2015), none of the reviewed studies created a new systematization for student misbehavior, and the SOMs does not answer the following questions: Does digitization provoke misbehavior that is not yet covered by the existing categories? Furthermore, how does digitization affect teachers’ misbehavior? As mentioned earlier in the framework of this review, models for classroom management already exist. It is not surprising that the findings of the different articles partly fit into these models: developing meta-awareness (Charles, 2012) fits into the choice theory model by Glasser (1986), letting students know what is acceptable behavior and what is not (Baker et al., 2016; Greenberg et al., 2014) corresponds with the assertive discipline model by Canter (2009), concrete strategies mentioned in Storch and Juarez-Paz (2019) relate to the model of Kounin (1970), and the often-mentioned rule-setting (Moltudal et al., 2019) can be found as one of the big five strategies in classroom management (Greenberg et al., 2014). The Classroom Disruption Protocol mentions the unallowed usage of cell phones, what is mentioned by Charles (2012) as well as parts of the SOMs by Li and Titsworth (2015) fits in parts of the major disruptions mentioned in the protocol. All the existing models refer to existing classroom practices but miss a connection to digitization. Furthermore, most models deal with classroom management in general and do not focus on the disruptions therewithin. An extension of an existing model into a digital setting or a model for disruptions in digital teaching is needed. It is conceivable, for example, that one or more specializations of the Classroom Disruption Protocol could represent a possible result here. Perhaps existing concepts are unsuitable for various reasons. This would make it necessary to develop a new type of model.

Research questions 4.1 to 4.3 deliver answers regarding what current research reports are the causes of disruptions in digital teaching and how to prevent them or intervene in relation to them. Among the results, the lack of student self-discipline is often mentioned as one of the main causes. This underlines the aforementioned focus of the research as being on the teacher’s perspective and is supported by the fact that all the studies used (pre-service) teachers as participants. Some studies included students as well, but the focus on the teacher’s perspective is clear. Studies that include the student’s perspective have the potential to deliver results that teacher-focused research cannot provide. Unfortunately, most of the analyzed articles are missing this potential opportunity. It is obvious that a deeper insight into disruptions from the student’s perspective will lead to a greater quantity of and interesting results.

Student and teacher behavior is often mentioned in this review. The leadership style of teachers is linked to classroom situations (like disruptions) and students’ learning outcomes (Solomon et al., 1964, p. 23). Since this review focuses on the aspect of disruptive classroom situations, there was no focus on teachers’ behavior strategies. Nevertheless, it must remain clear that there is no possibility of denying the connection between these two fields. Classical “core elements of General Didactics” (Zierer & Seel, 2012, p. 5) already understand interaction, and thus the behavior dimensions of those involved, as an essential part of interaction in teaching. The lack of findings with regard to specific disruptive situations in digital teaching suggests the need to investigate these further before developing possible prevention and intervention strategies. This review does not claim to predict whether there is a further field to explore in terms of research about behavior strategies, but since the idea that digitization can enhance existing concepts has already been mentioned, it can be assumed with optimism that future research will discover development potential here too.

Considering different perspectives on this field of (future) research seems to be a possible way of obtaining a more selective view of the three different types of digital teaching as well. It is noticeable that the majority of studies either focus on online learning or face-to-face learning. The blended learning approach, with its combined character, may be able to identify different disruptions from both groups. Keeping in mind that science has the obligation to expand existing research, this seems to be a good way of connecting the old and the new.

## Limitations

Since the selection of digital tools, the degree of influence and application as well as integration differ greatly depending on the subject, teacher, and type of school, it was difficult to assign this review to one specific area of ICT. In particular, further research may address relationships between types of disruptions and linked ICT areas like data security, requirements for educational platforms or databases. This may also lead to valuable insights on educationally influenced software developments.

While creating the search string for the literature research, it was impossible to include all existing digital tools. To avoid missing relevant articles, the search string included a lot of common combinations and big word groups, like the earlier mentioned terms of *behavior*, *technology*, and *classroom management*. Because of the lack of a uniform definition, this review had to be conducted on this very general level. As a consequence, the search term, which was equipped with general terms but nevertheless focused, sometimes also included articles that clearly missed the main perspective of the review. The review process therefore resulted in a significant reduction in the number of articles to be reviewed. This problem could not be circumvented due to the conceptual obstacles in the beginning, but an established standardized review-procedure was applied.

The large number of used designations created a need for a larger quantity of and more detailed exclusion criteria, while leaving wide fields of research open. During the reliability check process, the researchers stopped twice to adjust the criteria and to create a uniform understanding of all the terms. All the required understandings are based on the fact that there is a lack of uniform definitions in the first place. Nevertheless, it is possible that missing word combinations or the creation of a concrete understanding of specific terms “too late” in the review process led to very specific articles not being found. The suggestion regarding the development of uniform terminologies will help future research in this area.

## Future directions

Concerning one of the main findings of this review, further research should be directed at the field of digital teaching and the disruptions within. Observing digital teaching, interviewing students and teachers about their opinions, and keeping in mind that new generations of future teachers are growing up in a more digital world will create multiple opportunities for future research while keeping its practical relevance in mind. As already mentioned, research is still very focused on the teacher’s perspective. Having a more detailed view of disruptions in digital teaching from a student’s point of view seems promising.

The gathered information will be useful in terms of rethinking answers regarding causes of disruptions in digital teaching and possible prevention and intervention strategies. This will also open up new possibilities and potential for international comparison since a major part of the research (n = 8) in this field is located in the United States.

As mentioned, future research should focus on investigating concrete disruptive situations in digital teaching. For this, it will be important to have a clear understanding of what is considered to be a disruption and what is not. Since teaching and learning inevitably go together, opinions from teachers and students are important here. Future teachers are growing up in a more digital world and ICT, not only but especially in education, is becoming more and more important. One main goal of our research is to optimize teaching and teacher education. Therefore, adapting existing research (like the SOMs) or developing new models seems very promising. For example, an interview study with (prospective) teachers and students could provide basic knowledge that is necessary for designing a new model for classifying disruptions in digital teaching. Future research should also consider to investigate disruptions in specific situations and therefore have a look at actual practiced digital teaching.

## Conclusion

ICT has entered daily life, Digitization has entered education. With classroom management having been an important factor in terms of teaching for at least half a decade now and dealing with disruptive classroom situations being a part of successful classroom management, the possibilities and risks regarding the use of ICT within are getting more and more attention. The global Sars-CoV-2 pandemic forced educational systems worldwide into digital teaching faster than ever before and as part of an increased scientifically research on this field of interest, this review presented missing links to the important subject of classroom disruptions in digital teaching. Reviewing international, English-language articles about causes, prevention, and interventions with regard to disruptions in digital teaching from the last 20 years, this review shows the lack of precision when it comes to terminology and the absence of comprehension in terms of disruptive situations in a digital setting. The results point out that researchers are using multiple ways to approach this. The findings related to disruptions are still very general, largely teacher-sided, and not specific to digital teaching. With indications that digitization in teaching and learning has the possibility to enhance education, promising results will rely on more practically relevant and deeper research in the future.

## Data Availability

Not applicable.

## Appendix

**Table 4:** Overview of reviewed articles with findings on disruptions in digital teaching

Author(s):	Title:	Research design (RQ 2) and digital tools used:	Main findings, statistical findings, and quantitative data:
Baker et al. (2016)	A Model for Online Support in Classroom Management: Perceptions of Beginning Teachers	• Qualitative analysis of texts • n = 6 pre-service teachers’ • online support (videos, etc.)	• Different strategies in different situations for different participants • RQ 1: Disruptions, rule violations • RQ 4.2: Creating classroom routines; establishing positive relationships; professionality • RQ 4.3: Non-verbal actions; private conversations; closed questions on students’ behavior; early disengagement; quit taking it personally (QTIP)
Blundell et al. (2019)	Using Dual Systems Theory to Conceptualise Challenges to Routine When Transforming Pedagogy with Digital Technologies	• Qualitative interviews and focus groups • n = 6 teachers • Personalized digital learning	• Transforming practices with digital tools creates challenges • RQ 1: Digital technologies, digital learning • RQ 4.1: Digital tools themselves • RQ 4.2: Procedural routines; when teachers feel comfortable with their style of teaching
Boyaci (2010)	Pre-Service Teachers’ Views on Web-Based Classroom Management	• Qualitative interviews • n = 20 pre-service teachers • Web-based classroom management	• Web-based environment seems to be more important than classroom management in general, giving more responsibility to students • RQ 1: Web-based, education via internet, technical, software • RQ 4.1: Lack of/limited communication • RQ 4.2: Permanent communication, teachers’ participation
Bruhn et al. (2020)	The Effects of Data-Based, Individualized Interventions for Behavior	• Mixed-methods approach with questionnaire and app data analysis • n = 26 teachers and students • App	• Adaptions made through data-based individualized interventions are minimal • Students significantly (p < 0.001) increased their percentage of positive behavior (PBB) by 19.40% • RQ 1: Data-based, technology-based; behavioral interventions, challenging/problem behavior • RQ 4.3: Student self-monitoring via app
Charles (2012)	Cell Phones: Rule-Setting, Rule-Breaking, and Relationships in Classrooms	• Qualitative observations and interviews • n = 10 teachers • Cell phones	• Rule-setting is important when dealing with new technologies • RQ 1: Cell phones, social media, digital networks, mobile technologies; breaking rules, inappropriate behavior • RQ 4.1: Non-uniform rules • RQ 4.2: Developing a meta-awareness of discourse usage; clear understanding of rules
Heitink et al. (2017)	Eliciting Teachers’ Technological Pedagogical Knowledge	• Quantitative questionnaire • n = 29 teachers • Video vignettes (inside clips: multiple tools like PCs/laptops, interactive whiteboards, mobile phones, tablets)	• Teacher style affects student success, and therefore teachers need more practice in their use of digital tools to feel comfortable • RQ 1: Information and communication technology (ICT); Behavior supportive adaptive teaching
Ho (2004)	A Comparison of Australian and Chinese Teachers’ Attributions for Student Problem Behaviors	• Quantitative questionnaire • n = 473 teachers • Video vignettes	• Regardless of cultural background, teachers hold students responsible for misbehavior • Problem behaviors differ across cultural backgrounds [F (3,1401) = 17.56, p < 0.05) • RQ 1: Student/common/inappropriate problem behaviors • RQ 3: Different types of problems (daydreaming, talking, bullying, etc.) • RQ 4.1: Students’ effort/family/ability
Homer et al. (2018)	Comparing Digital Badges-and-Points with Classroom Token Systems: Effects on Elementary School ESL Students’ Classroom Behavior and English Learning	• Mixed-methods approach with pre/post-tests, observations, surveys, and text analysis • n = 120 students and teacher • Online token system	• Digital token system has positive impact on student behavior • RQ 1: Digital badges-and-point/token system; negative/off-task behavior • RQ 4.2: Using a digital token system • The percentage of “nearly all of the students” following instructions went from 0% to 37.5% (in reading classes) and from 0 to 80% (in speaking classes) • The percentage of “nearly all of the students” staying on task went from 0% to 18.8% (in reading classes) and from 0 to 70% (in speaking classes)
Hummel et al. (2015)	Collaboration Scripts for Mastership Skills: Online Game about Classroom Dilemmas in Teacher Education	• Mixed-methods approach with questionnaire and text analysis of feedback given • n = 19 pre-service teachers • Online game	• Pre-service teacher collaborations are useful for dealing with disruptions • RQ 1: Online; classroom management dilemmas
Judge et al. (2013)	The Use of Visual-Based Simulated Environments in Teacher Preparation	• Quantitative text analysis, focus groups, and questionnaire • n = 6 pre-service teachers • Virtual classroom simulation	• Virtual simulations can help pre-service teachers practice for dealing with disruptions but are still technically limited • All six participants had an increased usage of the “precise praise” strategy (from + 1 to + 9.33 in mean occurrences a session) • Two participants decreased their usage of the “planned ignoring” strategy (-2 and -8.67 mean occurrences a session), while the other four increased their usage of it (+ 1, + 5.33, + 10.33, and + 12.67) • RQ 1: Virtual technology/environment, mixed-reality; incompatible/disruptive/unengaged behavior, behavior management/disruptions
Kurz and Batarelo (2010)	Constructive Features of Video Cases to be used in Teacher Education	• Qualitative text analysis of feedback given • n = 27 pre-service teachers • Video vignettes	• Video cases can be used to evaluate classroom management strategies but not alone • RQ 1: Technology, video; students without discipline, disruptions
Li and Titsworth (2015)	Student Misbehaviors in Online Classrooms: Scale Development and Validation	• Mixed-methods approach (2 studies with surveys) with teachers and students • n varies • Online classroom	• Independent student learning tempts students to abuse technology. Communication with the teacher is important • All 15 items within the SOMs are loaded significantly (from 0.48 to 0.98) • Positive correlation between items and the number of online classes a student has taken: • Cheating (0.02); unallowed collaboration (0.03); plagiarism (0.13); inattentiveness (0.03); lack of critical thinking (0.03); procrastination (0.12); slacking over group work (0.06); slacking over individual work (0.04); nontextual aggressiveness (0.20*); aggressiveness toward teachers (0.13) • Negative correlation between items and number of online classes a student has taken: • Abusing technology (-0.06); aggressiveness toward classmates (-0.03); little communication with teacher (-0.19*); little communication with classmates (-0.01); no communication (-0.02) • RQ 1: Online, technology; misbehavior, (in-)appropriate behavior • RQ 3: Student Online Misbehavior scale (SOMs): • Seeking unallowed assistance (cheating, unallowed collaboration, plagiarism, abusing technology) • Internet slacking (inattentiveness, lack of critical thinking, procrastination, slacking over group work/individual work) • Aggressiveness (non-textual, toward teacher/classmates) • Lack of communication (no/little communication with teacher/classmates) * p < 0.05
Moltudal et al. (2019)	The Relationship between Teachers’ Perceived Classroom Management Abilities and Their Professional Digital Competence: Experiences from Upper Secondary Classrooms. A Qualitative Driven Mixed Method Study	• Mixed-methods approach with interviews, focus groups, observations, and surveys • n = 30 (qualitative); n = 2579 (quantitative) teachers, students, and school-related persons • Digital competence in general	• Classroom management is becoming more important when digital tools are used • RQ 1: Digital competence, information and communication technology (ICT); non-academic use, crime-control • RQ 4.1: Lack of student self-discipline; increased teacher digital competence • RQ 4.2: Rules
Muir et al. (2013)	Preparing Pre-Service Teachers for Classroom Practice in a Virtual World: A Pilot Study Using Second Life	• Qualitative text analysis of feedback • n = 8 pre-service teachers • Virtual classroom simulation	• Virtual simulations create opportunities to practice situations and simulate roles but are still technologically limited • RQ 1: computer-based, virtual classroom/world; classroom/student behavior (management)
Rosen and Beck-Hill (2012)	Intertwining Digital Content and a One-to-One Laptop Environment in Teaching and Learning: Lessons from the Time to Know Program	• Mixed-methods approach with assessment scores, school records, questionnaires, and observations • n = 496 teachers and students • Laptops	• One-to-one technology has potential to improve learning outcomes • Disciplinary issues in experimental classes decreased by 62.5% and in control classes by 15.4% • RQ 1: technology, computers; disciplinary issues
Storch and Juarez-Paz (2019)	Efficacy of Cell Phones within Instructional Design: A Professor’s Perspective	• Qualitative interviews • n = 10 teachers • PCs/laptops (online interviews); cell phones	• With preset behavioral expectations, benefits of cell-phone usage in classes are bigger than risks • RQ 1: cell phones; behavioral expectations • RQ 4.1: cell phones themselves • RQ 4.2: rules and policies; monitoring • RQ 4.3: giving warnings
